# Cortical Morphological Changes in Congenital Amusia: Surface-Based Analyses

**DOI:** 10.3389/fpsyt.2021.721720

**Published:** 2022-01-13

**Authors:** Xuan Liao, Junjie Sun, Zhishuai Jin, DaXing Wu, Jun Liu

**Affiliations:** ^1^Department of Radiology, The Second Xiangya Hospital of Central South University, Changsha, China; ^2^Department of Radiology, The Sir Run Run Shaw Hospital Affiliated to Zhejiang University School of Medicine, Hangzhou, China; ^3^Medical Psychological Center, The Second Xiangya Hospital of Central South University, Changsha, China; ^4^Clinical Research Center for Medical Imaging in Hunan Province, Changsha, China; ^5^Department of Radiology Quality Control Center, The Second Xiangya Hospital of Central South University, Changsha, China

**Keywords:** congenital amusia, surface-based morphology, music discrimination, middle frontal gyrus, pars triangularis gyrus, structural magnetic resonance imaging

## Abstract

**Background:** Congenital amusia (CA) is a rare disorder characterized by deficits in pitch perception, and many structural and functional magnetic resonance imaging studies have been conducted to better understand its neural bases. However, a structural magnetic resonance imaging analysis using a surface-based morphology method to identify regions with cortical features abnormalities at the vertex-based level has not yet been performed.

**Methods:** Fifteen participants with CA and 13 healthy controls underwent structural magnetic resonance imaging. A surface-based morphology method was used to identify anatomical abnormalities. Then, the surface parameters' mean value of the identified clusters with statistically significant between-group differences were extracted and compared. Finally, Pearson's correlation analysis was used to assess the correlation between the Montreal Battery of Evaluation of Amusia (MBEA) scores and surface parameters.

**Results:** The CA group had significantly lower MBEA scores than the healthy controls (*p* = 0.000). The CA group exhibited a significant higher fractal dimension in the right caudal middle frontal gyrus and a lower sulcal depth in the right pars triangularis gyrus (*p* < 0.05; false discovery rate-corrected at the cluster level) compared to healthy controls. There were negative correlations between the mean fractal dimension values in the right caudal middle frontal gyrus and MBEA score, including the mean MBEA score (*r* = −0.5398, *p* = 0.0030), scale score (*r* = −0.5712, *p* = 0.0015), contour score (*r* = −0.4662, *p* = 0.0124), interval score (*r* = −0.4564, *p* = 0.0146), rhythmic score (*r* = −0.5133, *p* = 0.0052), meter score (*r* = −0.3937, *p* = 0.0382), and memory score (*r* = −0.3879, *p* = 0.0414). There was a significant positive correlation between the mean sulcal depth in the right pars triangularis gyrus and the MBEA score, including the mean score (*r* = 0.5130, *p* = 0.0052), scale score (*r* = 0.5328, *p* = 0.0035), interval score (*r* = 0.4059, *p* = 0.0321), rhythmic score (*r* = 0.5733, *p* = 0.0014), meter score (*r* = 0.5061, *p* = 0.0060), and memory score (*r* = 0.4001, *p* = 0.0349).

**Conclusion:** Individuals with CA exhibit cortical morphological changes in the right hemisphere. These findings may indicate that the neural basis of speech perception and memory impairments in individuals with CA is associated with abnormalities in the right pars triangularis gyrus and middle frontal gyrus, and that these cortical abnormalities may be a neural marker of CA.

## Introduction

Music is a fundamental element in interpersonal and social communication. However, 1.5–4% of the general population exhibits lifelong impairments in music production, perception, and memory (1, 2), in the absence of any brain damage, hearing loss, and cognitive deficits (3, 4). This neurogenetic mental condition is known as congenital amusia (CA) (3, 4). Behavioral studies have demonstrated that CA is a musical pitch-processing disorder that manifests as deficits in pitch perception and pitch memory (5). Individuals with CA are unable to perceive fine-grained pitch changes, which means that they cannot detect dissonances and out-of-key tones when they (or others) sing out of tune (6). The processing of musical rhythm, memory, and emotions can also be affected in CA (4). This disorder is usually diagnosed using the Montreal Battery of Evaluation of Amusia (MBEA), which assesses the temporal and melodic dimension of music, as well as musical memory. The temporal dimension includes rhythm and meter subscales, and the melodic dimension includes scale, contour, and interval subscales (7).

Several imaging studies have been conducted to understand the neurobiological mechanisms underlying pitch disorders in people with CA. Previous functional magnetic resonance imaging (fMRI) studies have indicated that abnormal activity in the right frontotemporal network (8, 9) and/or a dysfunction of the auditory cortex (10, 11) play important roles in music perception and memory of individuals with CA. A diffusion tensor imaging study has also shown that individuals with CA have abnormally higher diffusivity indices the right inferior/superior longitudinal fasciculus and the right inferior frontal-occipital fasciculus, which indicates that the fronto-temporal pathway is impaired in patients with CA (12). Some structural MRI studies have also used voxel-based morphometry (VBM) to validate that CA is a neurodevelopmental disorder that is accompanied by cortical abnormalities (10, 13–16). For instance, Hyde et al. found that individuals with CA had a reduced white matter volume in the right IFG (16); the same authors also found that those with CA have a thicker cortex in the right IFG and the right auditory cortex (14). Similarly, Albouy et al. confirmed that individuals with CA have morphological brain abnormalities, namely, in white and gray matter volume in the right superior temporal gyrus and right IFG (10). However, a structural MRI study with a larger sample size showed that those with CA had a reduction in gray matter volume in the left superior temporal sulcus and posterior IFG (13). Therefore, it is still controversial as to whether the neuroanatomical abnormalities associated with CA involves the left or right brain hemisphere. This problem may need to be addressed using novel methods.

Furthermore, these anatomical abnormalities associated with amusia cannot fully explain the clinical manifestations of CA. It has been reported that individuals with CA have impairments that extend to other domains, including speech perception (17–22), emotion (23–26), memory (10, 27, 28), and visual perception (29). For example, Jiang et al. found that healthy controls (HCs) elicited a larger P600 and smaller N100 in response to inappropriate prosody compared with appropriate prosody, while no such differences in either the N100 or the P600 component were found in those with CA; this indicates that CA may affect intonation processing during speech comprehension (17). One explanation for these speech perception disorders in CA is the pitch processing abnormality (30). Some researchers believe pitch to be an essential element of auditory processing in music and language, and one of the cues used to decipher emotion (30). However, in the absence of anatomical abnormalities, the claim that those with CA also exhibit other behavioral impairments remains controversial. Therefore, it is important to investigate cortical morphological changes using a novel approach; doing so may illuminate the anatomical mechanism underlying other behavioral abnormalities observed in CA.

Surface-based morphology (SBM) and VBM are common methods by which to learn about structural abnormalities (31). VBM is one of the most commonly used methods to analyze brain structures, but it adopts voxel-based registration to reduce individual variability, which may lead to registration artifacts (32). More importantly, VBM takes the highly variable folding pattern of the brain into consideration, which may further reduce the accuracy of alignment (33). These conditions may lead to uncorrected gray and white matter, and an inability to obtain true volume changes caused by disease (34). Conversely, SBM applies an alternative approach to register by matching the gyral and sulcal geometry to an inflated spherical atlas, which can reduce the potential misalignment induced by complex folding patterns and/or global volume differences (35–37). Furthermore, SBM is able to produce more cortical parameters than VBM, which can reflect cortical morphological changes in a more multidimensional (38), sensitive (39), and accurate (40) way. Four parameters of each hemisphere can be obtained using SBM analysis, including cortical thickness, fractal dimension, sulcal depth, and gyrification index. Cortical thickness is calculated by the distance between the inner (boundary between white and gray matter) and outer (boundary between gray matter and cerebrospinal fluid) cortical surfaces (41). Fractal dimension is a measure of shape complexity, and it has been considered as a combination of the frequency of cortical folding, sulcal depth, and the convolution of gyral shape (42). The gyrification index is the ratio between the pial surface and the outer smoothed surface of the cortex, and indicates the amount of cortex in the sulcal folds relative to the outer visible cortex (43). Sulcal depth is calculated based on the Euclidean distance between the convex hull and the central surface (44).

In the current study, we systematically investigated brain structural abnormalities in individuals with CA and HCs using SBM. We hypothesized that individuals with CA would show alterations in SBM metrics in brain regions associated with language, memory, and/or other domains. We also hypothesized that some of these abnormalities would be correlated with clinical parameters, such as the MBEA score.

## Materials and Methods

### Participants

**Figure 1:**
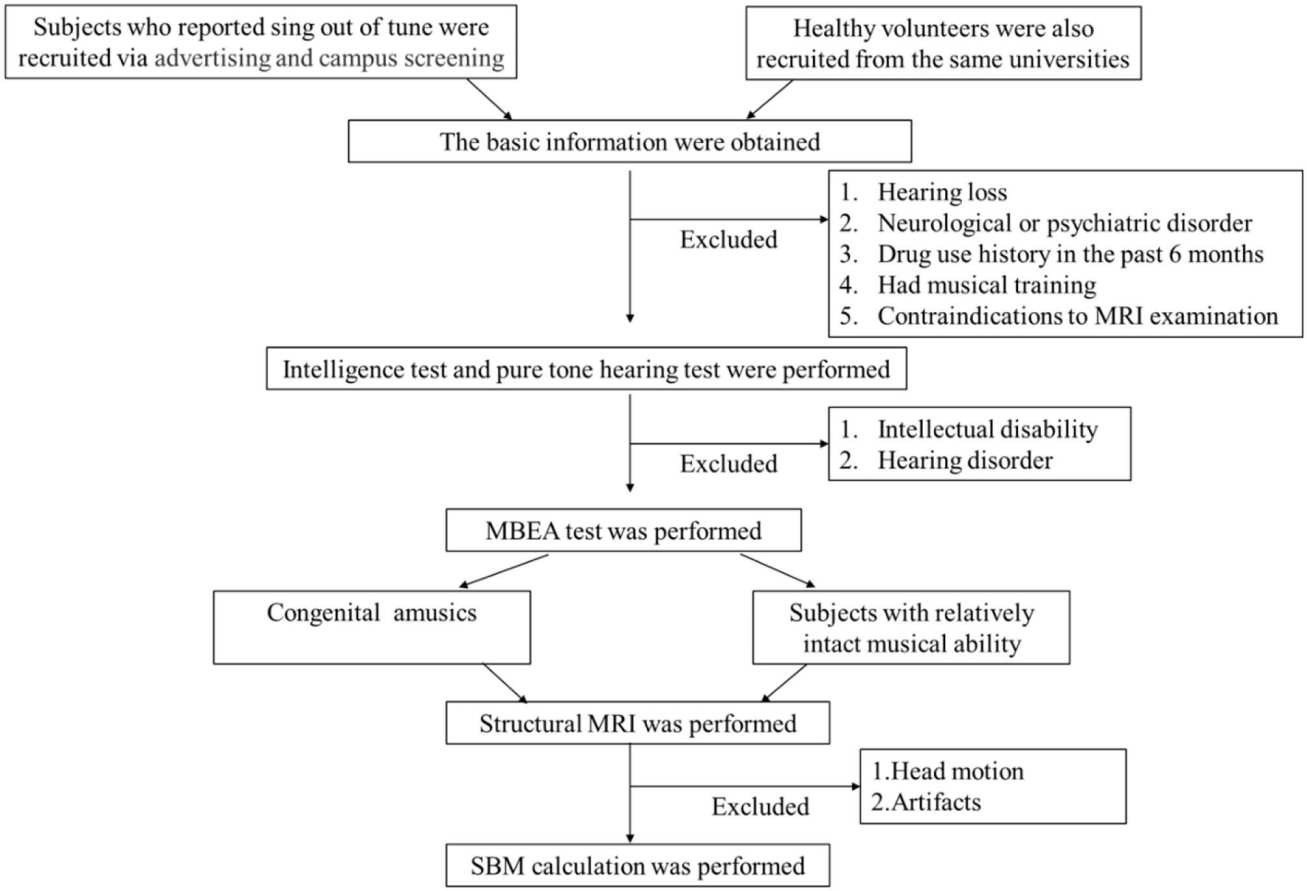
Flowchart of patient selection. MRI, magnetic resonance imaging; MBEA, the Montreal Battery of Evaluation of Amusia; SBM, surface-based morphology.

We recruited Chinese college students in Changsha, Hunan Province, China, who self-reported singing out of tune. Participants were recruited *via* advertisements and campus screening between November 2018 and August 2019. First, we conducted a structured clinical interview to collect basic information (age, sex, handedness, health conditions, and years of education). All subjects spoke Mandarin and were right-handed. Second, the Wechsler Intelligence Scale and a pure tone audiometary test were used to exclude the possibility of hearing and/or intellectual deficits. The specific inclusion criteria were as follows: (1) a Wechsler Intelligence Scale score of more than 85, which indicates normal intelligence, and (2) the ability to hear at least 25 decibels, as measured by pure tone audiometry. The exclusion criteria were as follows: (1) hearing loss, (2) the presence of a neurological or psychiatric disorder, (3) drug use history in the past 6 months, (4) had received musical training, and (5) contraindications to MRI examination. The HCs were recruited from the same universities as the individuals with CA; they fulfilled the same exclusion criteria and were matched to the CA group for age, sex, handedness, and education. Third, all participants completed the MBEA face to face to assess ability in the temporal dimension (rhythm and meter) and melodic dimension (scale, contour, and interval), and in musical memory (7). We then calculated each individual's score of each MBEA subtest (rhythm, meter, scale, contour, interval, and memory), the global MBEA score (sum of the scores of the six subtests), and mean MBEA score (the global MBEA score divided by 6). If an individual's average MBEA score was < two standard deviations from the normal control mean, they were considered as having CA (7). In addition, some studies suggested that the MBEA test had a potential misclassification, which could be attributed to a high rate in Type II error (45–47). To avoid this, signal detection theory (SDT) analysis was performed in our study to confirm the results of MBEA test. Finally, all subjects underwent MRI scanning at the Second Xiangya Hospital of Central South University. The recruitment process is shown in Figure 1. The study was approved by The Ethics Committee of the Second Xiangya Hospital, Central South University, and written informed consent was received from all participants prior to their participation in the study.

### Signal Detection Theory Analysis (SDT)

The MBEA test was made up of six subtests, and each of the subtests contains 30 pairs of melodies. For the subtests of the MBEA, there are two stimulus classes associated with same vs. different, march vs. waltz, and new vs. old. When a stimulus is presented, subjects were needed to judge whether the stimulus is from Class A (e.g., same, waltz, or new) or Class B (e.g., different, march or old) (7). In our study, each subject's response to each pair of melodies was collected. Then, the hit rate and false alarm rate were calculated, cf. (1, 48). A hit refers to the different, march, or old melody was accurately classified into Class B. A false alarm means that the same, waltz, or new melody was incorrectly classified into Class B (45, 46) (Table 1). Next, the d' value and c value measured by SDT were calculated, cf. (2, 3, 49). The d' value was mainly used to reflect the sensitivity performance of test. The higher the d' value, the better the subject discriminates between stimuli. A d' value of 0 refers to a subject inability to discriminate between stimuli (46). The c value was used to evaluate participants' response bias. Positive c values mean that subjects generally tend to respond “same,” while negative c values imply that individuals are inclined to respond “different” (50). Finally, the mean minus one standard deviation of global d' value in the HCs was used as a cutoff value to distinguish CA from HCs to verify the results of MBEA test (46).

1. Hit rate = hit number/(hit number + miss number); false alarm rate = false alarm number/(false alarm number + correct rejection number)
2. d' = Z(HR)-Z(FAR)
3. c = −0.5·[Z(HR)+Z(FAR)]

**Table 1:** Overview of stimulus types and possible response.

**Stimulus pair**	**Response**	
	**Different, march, or old**	**Same, waltz, or new**
Different, march, or old	Hit (H)	Miss (M)
Same, waltz, or new	False alarm (FA)	Correct rejection (CR)

### Image Acquisition

All MRI data were collected on a 3.0-T Siemens Skyra MRI scanner (Magnetom Skyra, Siemens, Munich, Germany) using a 20-channel head coil. Participants' heads were immobilized in the scanner with foam cushions and earplugs were worn to reduce the noise. Our protocol included a T1-weighted high-resolution three-dimensional sagittal Magnetization Prepared Rapid Acquisition Gradient Echo sequence with the following acquisition parameters: repetition time = 1,900.0 ms, echo time = 2.03 ms, flip angle = 9°, 176 slices, slice thickness = 1 mm, slice spacing = 1 mm, field of view = 256 × 256 mm, acquisition matrix = 64 × 64 mm, voxel size = 1.0 × 1.0 × 1.0 mm.

### Surface-Based Morphometry Analysis

Image data processing was performed using Statistical Parametric Mapping 8 (SPM8, www.fil.ion.ucl.ac.uk) and the Computational Anatomy Toolbox (CAT12, www.neuro.uni-jena.de/cat) in the MATLAB environment (R2013b, www.mathworks.com). All images were transformed to a Nifti-format using dcm2nii (http://www.nitrc.org/projects/mricrogl) and were visually inspected for structural abnormalities, artifacts, and apparent head motion before preprocessing. Next, all images were manually reoriented to have the same point of origin (anterior commissure) and spatial orientation.

The SBM processing was also performed using CAT12, which is based on the SPM8 software in the MATLAB environment. The SBM processing included the following steps: (1) T1-weighted Magnetization Prepared Rapid Acquisition Gradient Echo images were normalized and further segmented into gray matter volume, white matter volume, and cerebrospinal fluid volume. (2) In CAT12, projection-based thickness was used to estimate cortical thickness and create the central cortical surface for the left and right hemispheres (51). Surface reconstruction included topology correction (35), spherical inflation (36), and spherical registration (37). (3) Additional surface parameters, such as gyrification, cortical complexity, and sulcal depth, were extracted using CAT12. (4) All data were resampled into the template space to analyze surface parameters and were smoothed. Thickness meshes were smoothed with a 15-mm Gaussian kernel, and a 20-mm kernel was used for the other surface parameters.

### Statistical Analysis

Statistical analysis was performed using SPSS v25.0 (IBM Corp, Armork, New York, USA). The Shapiro–Wilk test was used to assess normality. Unpaired Student's *t*-tests were used to assess between-group differences for normally distributed data (e.g., age, years of education, and MBEA scores). The Fisher's exact test was used to perform between-group comparisons of categorical variables (e.g., sex). To eliminate artifacts and slight head motion, such as pulsatile effects from the vasculature and partial volume effects in boundary regions, subjects with any slight head motion (a translation movement of more than 1.5 mm, or a rotation more than 1.5°) were excluded. The cortical thickness and other surface parameter maps of the left and right hemispheres were separately assessed within a brain mask using voxel-wise two-sample *t*-tests in SPM8 and the CAT12 toolbox. All statistical maps were assigned thresholds at *p* < 0.001 (voxel level), and the false discovery rate was corrected to *p* < 0.05 at the cluster level for multiple comparisons. The surviving clusters are reported in the following results. We also used a regions-of-interest tools module in the CAT12 toolbox to extract the mean values within regions of interest defined using the Desikan–Killiany–Tourville atlases. The mean cortical thickness and additional surface parameters of clusters with statistically significant between-group differences were obtained. Finally, Pearson's correlation analysis was applied to assess the correlation of the MBEA global score and subscores with surface parameters. Statistical significance was defined as a *p*-value < 0.05.

## Results

### Demographic and Montreal Battery of Evaluation of Amusia Performance

After checking MRI images and head motion, eight subjects were excluded (three from the CA group and five from the HC group). One participant from the HC group was excluded due to slight head motion, and all other excluded participants were excluded due to artifacts. Finally, 15 participants with CA (with a global MBEA score of 119.87 ± 8.96, ranging from 98 to 129) and 13 HCs (with a global MBEA score of 165.46 ± 5.87, ranging from 158 to 174) were included in our study. The average MBEA score ranged from 16.33 to 21.5 in the CA group, and from 26.33 to 29 in the HC group. Table 2 presents the demographic information of the CA and HC groups. There were no significant between-group differences in age (*p* = 0.274), sex (*p* = 0.266), or years of education (*p* = 0.892). There were significant between-group differences in each MBEA subscore, whereby the CA group had significantly lower scores on all MBEA subtests compared to the HCs (*p* = 0.000).

**Table 2:** Characteristics of the congenital amusia and healthy control groups.

	**Congenital amusics**	**Healthy controls**	***p*-value**
Number (male/female)	15 (10/5)	13 (5/8)	0.266^†^
Age (years)	18.667 ± 0.817	19.076 ± 0.954	0.274^*^
Education (years)	13.200 ± 0.414	13.230 ± 0.439	0.892^†^
**Melodic discrimination**			
Scale score	19.067 ± 2.576	27.539 ± 1.664	0.000^*^
Contour score	20.867 ± 3.314	28.385 ± 1.660	0.000^*^
Interval score	19.267 ± 2.658	27.769 ± 1.691	0.000^*^
**Temporal discrimination**			
Rhythmic score	20.133 ± 1.922	27.462 ± 1.198	0.000^*^
Meter score	19.333 ± 4.638	26.077 ± 2.900	0.000^*^
**Memory score**	21.200 ± 3.877	28.231 ± 1.739	0.000^*^
Average MBEA score	19.977 ± 1.494	27.577 ± 0.978	0.000^*^

*Fisher's exact test*

(†) *and two-sample t-tests*

(^*^) *were used to test between-group differences in categorical and continuous variables, respectively*.

*MBEA, Montreal Battery of Evaluation of Amusia*.

### Scoring With Signal Detection Theory Analysis

**Figure 2:**
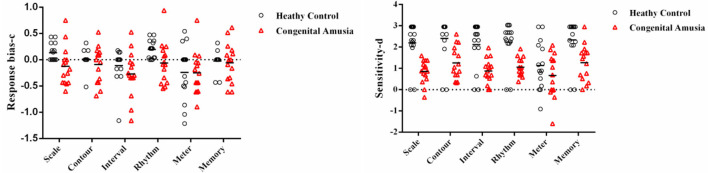
Signal Detection Theory scores (d' and c) plotted in per subtest. The categorization based on PC scores. The *c* value of scale subtest (*p* = 0.018) and rhythm subtest (*p* = 0.020) in the congenital amusia group is lower than the healthy controls. The d' value of the scale subtest (*p* = 0.000), contour subtest (*p* = 0.000), interval subtest (*p* = 0.000), rhythm subtest (*p* = 0.000), and memory subtest (*p* = 0.000) in the congenital amusia group is lower than the healthy controls.

SDT analyses were conducted in order to verify the results of the MBEA test. The mean and standard deviation of d' and c were calculated for every subtest. The skew and kurtosis of d' suggested that the d' value on every subtest basically is normally distributed (Table 3). There were statistical differences in the c value between CA and HCs in the scale subtest (*t* = 2.520, *p* = 0.018) and rhythm subtest (*t* = 2.474, *p* = 0.020) (Figure 2; Table 4). There were also statistical differences in d' value between CA and HCs, including the scale subtest (*t* = 9.302, *p* = 0.000), interval subtest (*t* = 6.678, *p* = 0.000), contour subtest (*t* = 6.740, *p* = 0.000), rhythm subtest (*t* = 10.506, *p* = 0.000), and memory subtest (*t* = 5.671, *p* = 0.000) (Figure 2; Table 5). Besides, the global d' score and global c score were calculated. The mean and standard deviation of global d' score was 0.999 ± 0.310 in the CA group and 2.865 ± 0.575 in the HC group; the mean and standard deviation of global c score was −0.156 ± 0.246 in the CA group and 0.045 ± 0.249 in the HC group. There were statistical differences between CA and HCs in the in the global d' score (*t* = 10.888, *p* = 0.000) and global c score (*t* = 2.132, *p* = 0.043). Finally, the mean minus one standard deviation of global d' value in the HC group (2.29 = 2.865–0.575) was taken as cutoff to identify CA. On this diagnostic criteria, 13 subjects were diagnosed as HCs and 15 were diagnosed with CA, which was similar with the results of MBEA. Therefore, these subjects were included in subsequent studies.

**Table 3:** Mean and SD of d' and c measured by signal detection theory.

		**Scale**	**Contour**	**Interval**	**Rhythm**	**Meter**	**Memory**	**Average**	**Pitch average**
d'	Mean	1.635	1.957	1.605	1.760	0.959	1.928	1.865	1.761
	SD	0.977	0.969	0.998	0.862	1.095	0.971	1.046	1.182
	z skew	−0.195	−0.464	−0.021	−0.009	−0.200	−0.579	0.458	0.371
	z kurtosis	−0.978	−1.349	−1.387	−1.476	−0.072	−0.852	−0.959	−1.457
c	Mean	0.004	−0.044	−0.205	0.073	−0.263	−0.444	−0.063	−0.151
	SD	0.320	0.284	0.395	0.335	0.468	0.302	0.263	0.397

*MBEA, Montreal Battery of Evaluation of Amusia; SD, standard deviation*.

**Table 4:** c value measured by SDT in the congenital amusia group and healthy control group.

	**Congenital amusics**	**Healthy controls**	***p*-value**
Scale	−0.126 ± 0.366	0.153 ± 0.171	0.018^*^
Contour	−0.091 ± 0.345	0.011 ± 0.189	0.349
Interval	−0.270 ± 0.432	−0.130 ± 0.349	0.356
Rhythm	−0.061 ± 0.391	0.227 ± 0.163	0.020^*^
Meter	−0.252 ± 0.399	−0.277 ± 0.555	0.891
Memory	−0.058 ± 0.374	−0.029 ± 0.202	0.801
Global	−0.156 ± 0.246	0.045 ± 0.249	0.043^*^

*SDT, signal detection theory*.

p* *< 0.05, which indicated that there was a statistical difference in the c value between the two groups*.

**Table 5:** d' value measured by SDT in the congenital amusia group and healthy control group.

	**Congenital amusics**	**Healthy controls**	***p*-value**
Scale	0.852 ± 0.546	2.537 ± 0.384	0.000^*^
Contour	1.253 ± 0.758	2.769 ± 0.328	0.000^*^
Interval	0.877 ± 0.576	2.444 ± 0.654	0.000^*^
Rhythm	1.050 ± 0.430	2.578 ± 0.321	0.000^*^
Meter	0.660 ± 1.020	1.305 ± 1.113	0.125
Memory	1.267 ± 0.836	2.689 ± 0.367	0.000^*^
Global	0.999 ± 0.310	2.865 ± 0.575	0.000^*^

*SDT, signal detection theory*.

p* *< 0.05, which indicated that there was a statistical difference in the d value between the two groups*.

### Surface-Based Morphometry Results

**Figure 3:**
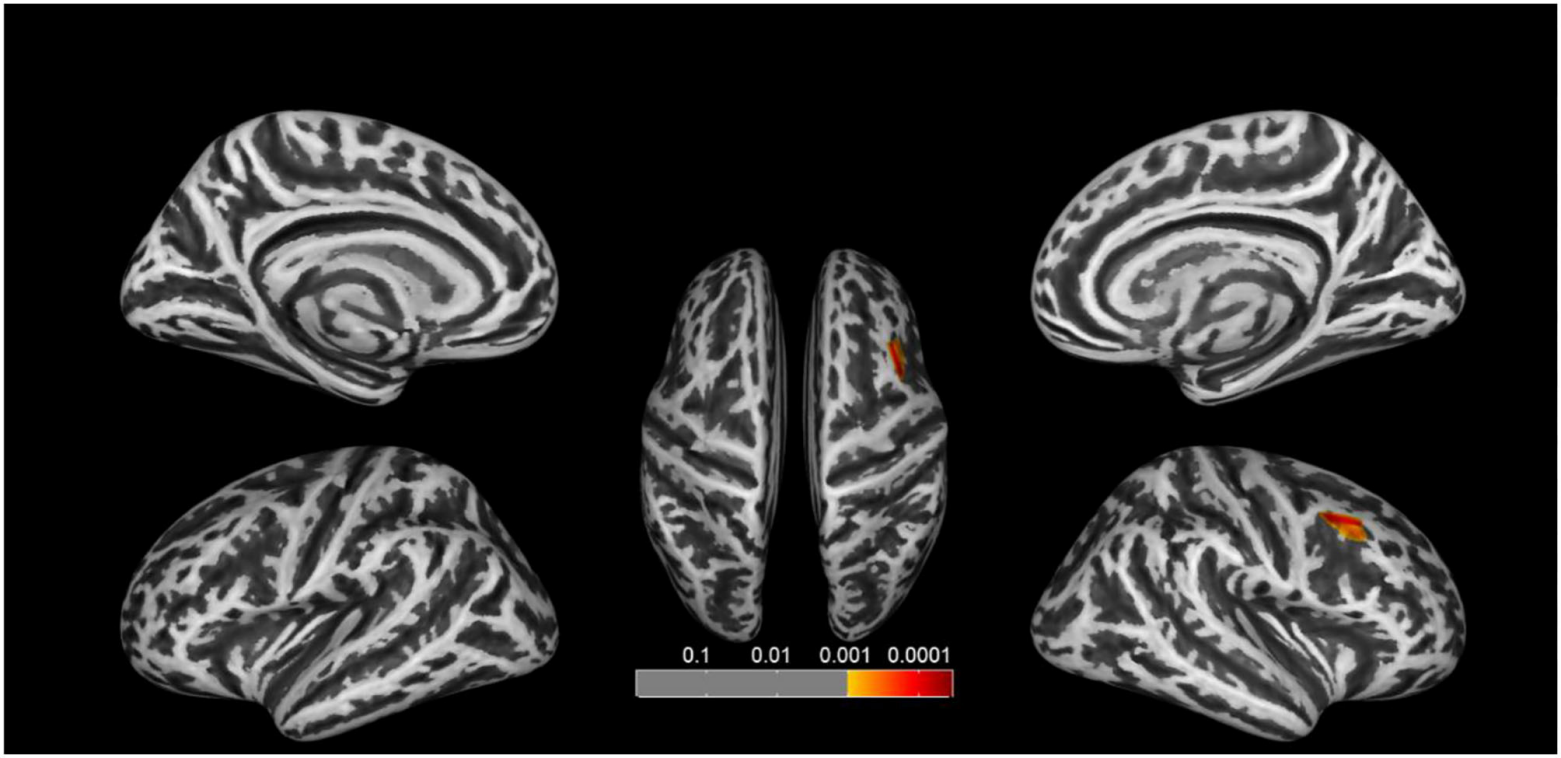
Clusters that significantly varied in terms of fractal dimension in the CA group vs. the HC group. The congenital amusia group had a significantly higher fractal dimension value in the right caudal middle frontal gyrus compared to healthy controls (*p* < 0.05; false discovery rate-corrected at the cluster level).

**Figure 4:**
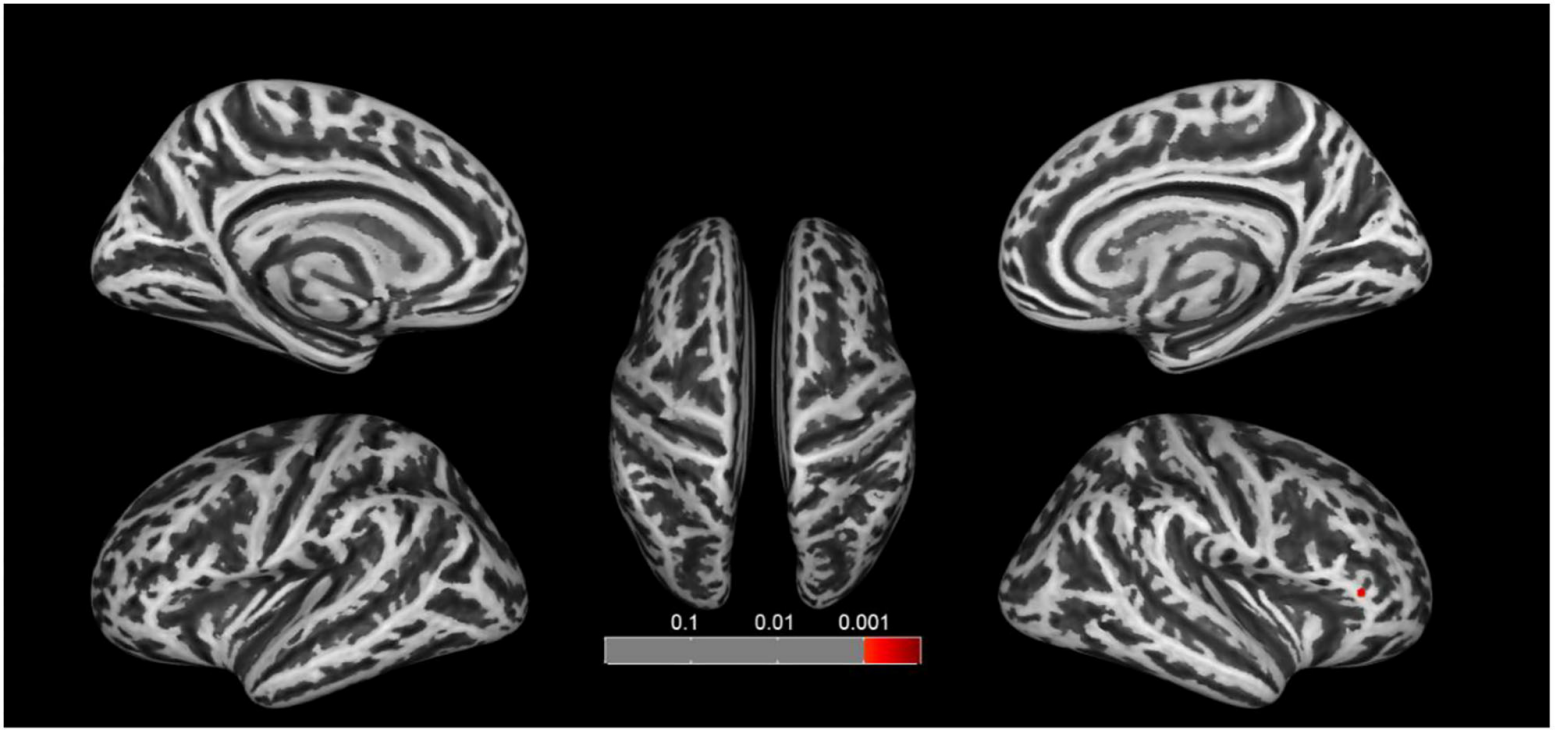
Clusters that significantly varied in terms of sulcal depth in the CA group vs. the HC group. Subjects with congenital amusia had a significantly lower sulcal depth in the right pars triangularis compared to healthy controls (*p* < 0.05; false discovery rate-corrected at the cluster level).

**Figure 5:**
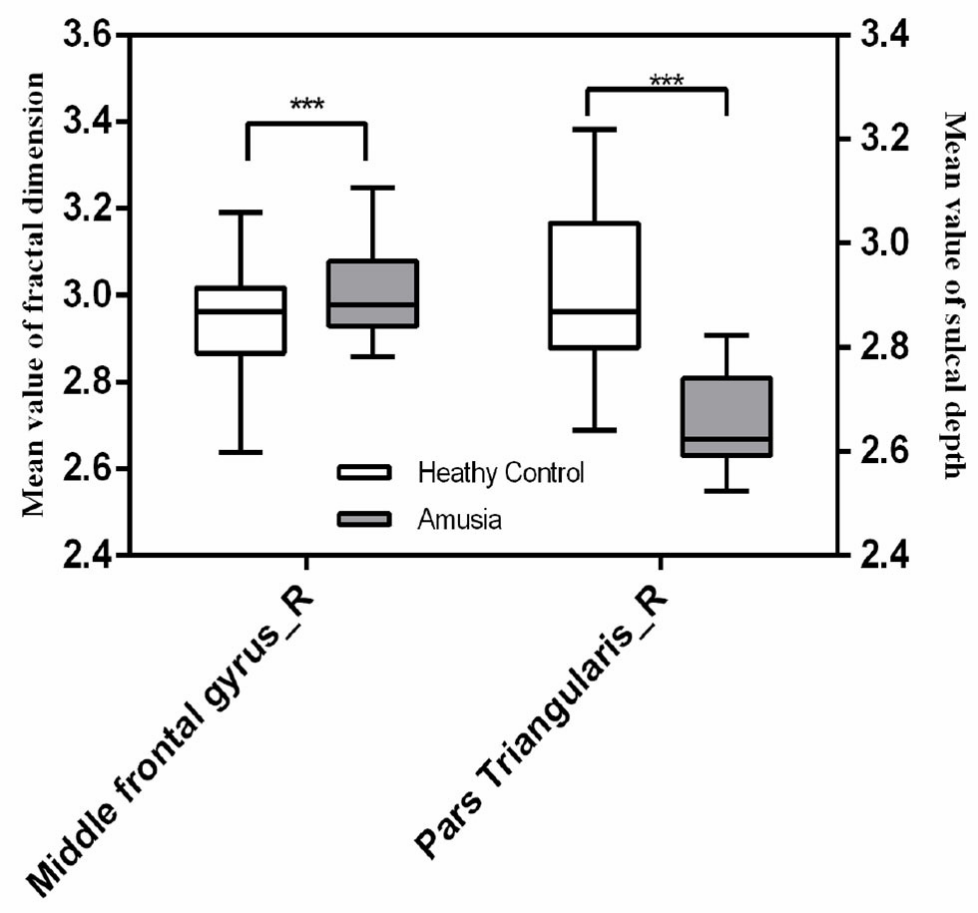
The mean fractal dimension value in the right caudal middle frontal gyrus and sulcal depth in the right pars triangularis in the two groups. The right caudal middle frontal gyrus exhibited a significant increase in the mean fractal dimension value in the congenital amusia group, and the right pars triangularis gyrus showed a significant decrease in the mean fractal dimension value in the congenital amusia group. ***p < 0.001.

We found significant increases of fractal dimension in the right caudal middle frontal gyrus (MFG; Figure 3) and a decrease of sulcal depth in the right pars triangularis gyrus (Figure 4) in the CA group compared to the HC group (*p* < 0.05; false discovery rate-corrected at the cluster level). The cluster size and peak value in the two brain regions are shown in Table 6. In addition, as shown in Figure 5, there were significant between-group differences in the mean sulcal depth in the right pars triangularis and the mean fractal dimension value in the right caudal MFG. Furthermore, the mean sulcal depth of significantly decreased clusters in the right pars triangularis areas were significantly lower in the CA group than in the HC group (*p* = 0.001), and the mean fractal dimension values of significantly increased clusters in the right caudal MFG were significantly larger in the CA group (*p* = 0.002).

**Table 6:** Abnormal structure located by cluster in the CA group assessed by surface-based morphology analysis.

**Overlap of atlas region**	**Cluster size**	***p-*value (corrected)**
100% Pars triangularis_R	51	0.00047
100% Caudal middle frontal_R	630	0.00009

*The significance level was set at p < 0.05 corrected for false discovery rate at the cluster level. R, right; CA, congenital amusia*.

### Correlations Between Musical Ability and Cortical Parameters

**Figure 6:**
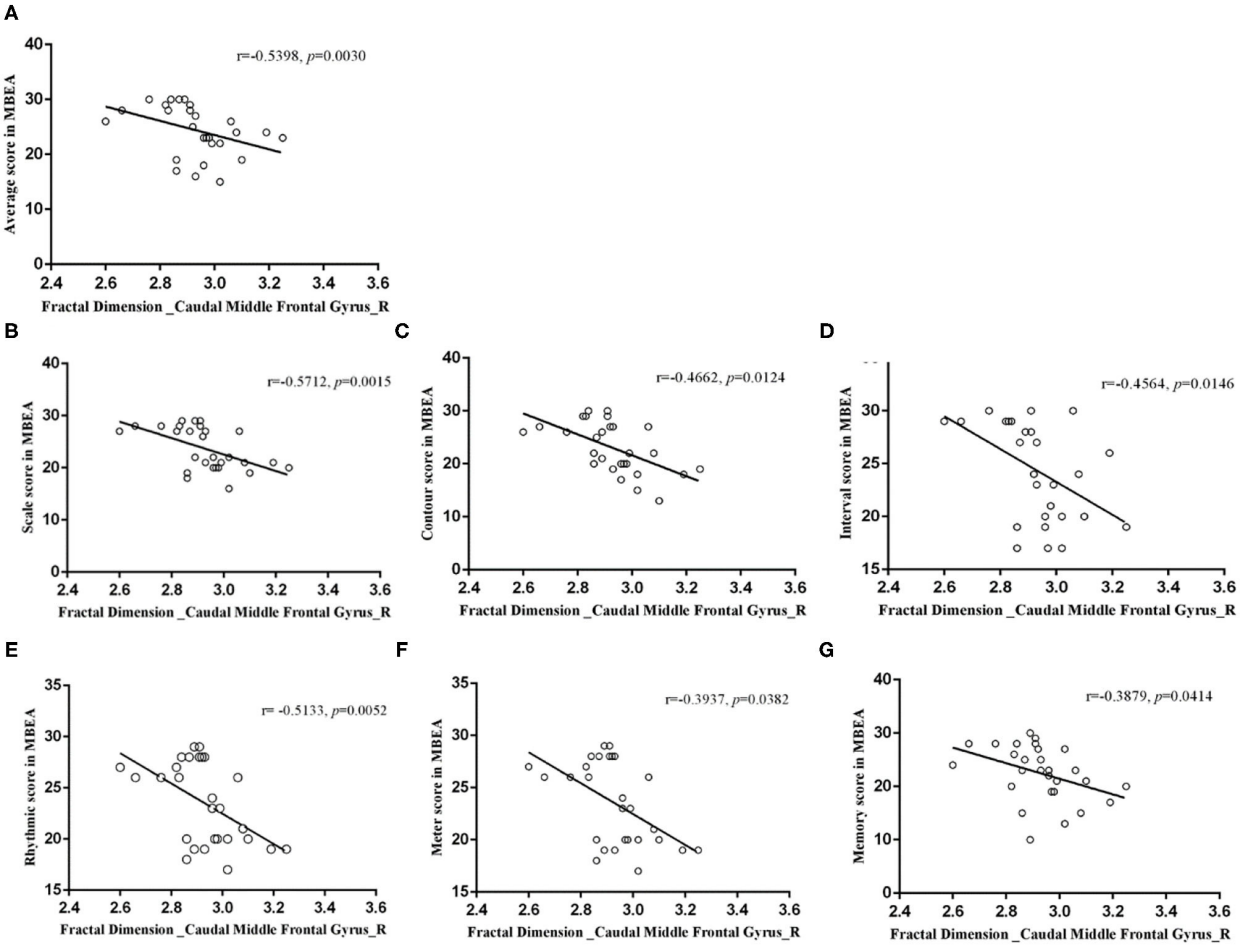
Correlations between the mean fractal dimension value in the right caudal middle frontal gyrus and MBEA test and subtest scores. In the right caudal middle frontal gyrus, the mean fractal dimension value was negatively correlated with the mean MBEA score **(A)**, pitch score **(B)**, contour score **(C)**, interval score **(D)**, rhythmic score **(E)**, meter score **(F)**, and memory score **(G)**.

**Figure 7:**
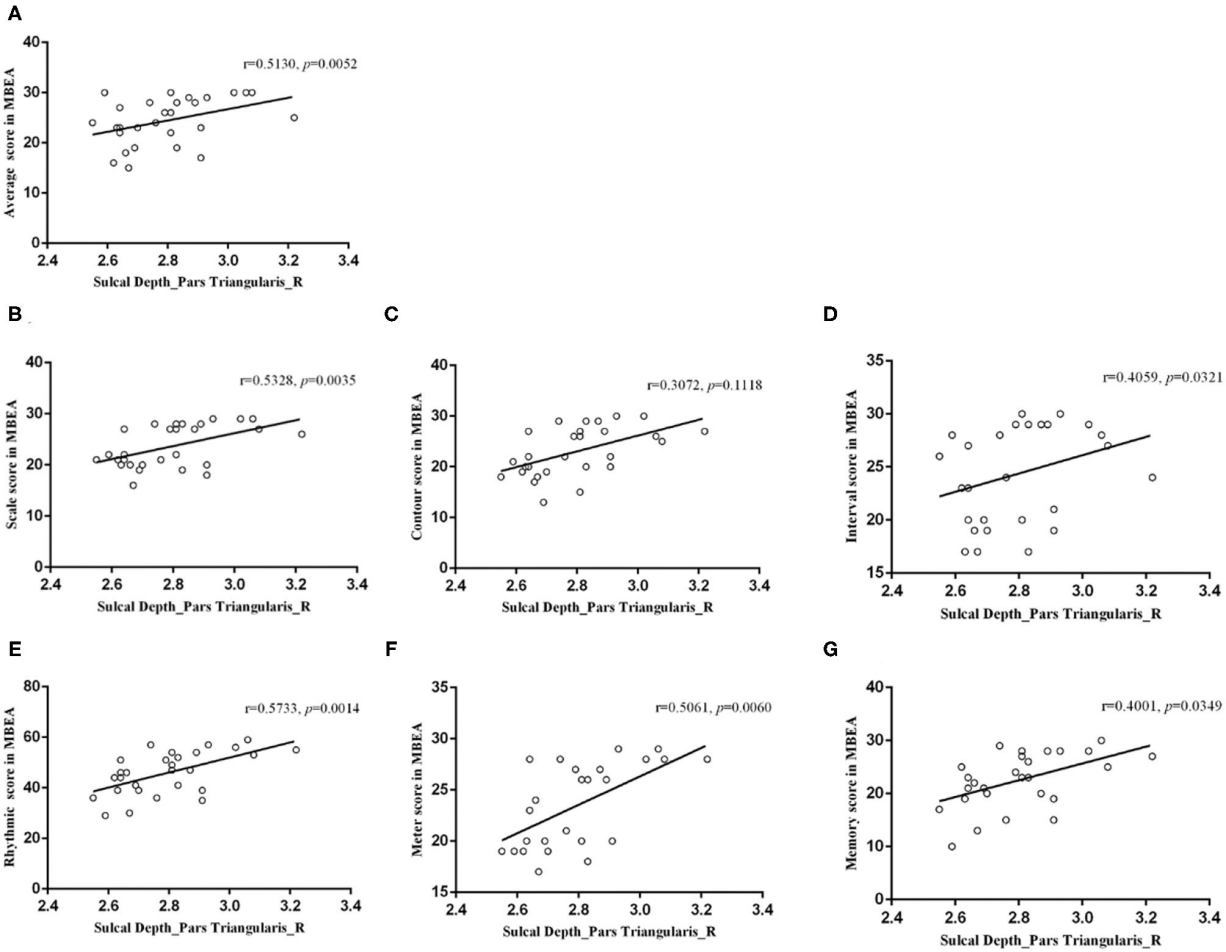
Correlations between the mean sulcal depth in the right pars triangularis and the MBEA test and subtest scores. In the right pars triangularis gyrus, the mean sulcal depth was positively correlated with the mean MBEA score **(A)**, pitch score **(B)**, interval score **(D)**, rhythmic score **(E)**, meter score **(F)**, and memory score **(G)**.

In the right caudal MFG, the mean value of fractal dimension was negatively correlated with the mean MBEA score (*r* = −0.5398, *p* = 0.0030), scale score (*r* = −0.5712, *p* = 0.0015), contour score (*r* = −0.4662, *p* = 0.0124), interval score (*r* = −0.4564, *p* = 0.0146), rhythmic score (*r* = −0.5133, *p* = 0.0052), meter score (*r* = −0.3937, *p* = 0.0382), and memory score (*r* = −0.3879, *p* = 0.0414; Figure 6). In the right pars triangularis gyrus, the mean sulcal depth was positively correlated with the mean MBEA score (*r* = 0.5130, *p* = 0.0052), scale score (*r* = 0.5328, *p* = 0.0035), interval score (*r* = 0.4059, *p* = 0.0321), rhythmic score (*r* = 0.5733, *p* = 0.0014), meter score (*r* = 0.5061, *p* = 0.0060), and memory score (*r* = 0.4001, *p* = 0.0349) (Figure 7).

## Discussion

The present structural MRI study was conducted to identify regions with cortical feature abnormalities at the vertex-based level in individuals with CA. The CA group exhibited a lower sulcal depth in the right pars triangularis gyrus and higher fractal dimension in the right caudal MFG. Furthermore, the MBEA score and subscores were negatively correlated with the mean fractal dimension value in the right caudal MFG. There was also a positive correlation between performance on the MBEA (except for the contour subtest) and sulcal depth in the right pars triangularis gyrus.

To our knowledge, this is the first study to investigate structural abnormalities using SBM in individuals with CA, and is also the first to report structural abnormalities in the right MFG in patients with CA. The MFG, which is located anterior to the premotor cortex and posterior to the dorsolateral prefrontal cortex (52), is an important cortical region that is involved in working memory (53, 54). A previous study revealed that the MFG is the core region underlying working memory (55). Indeed, the MFG and dorsolateral prefrontal cortex have been consistently reported to be involved in working memory encoding, storage, maintenance, and executive control (56–58). In our study, we found that the mean fractal dimension value in the MFG was higher in the CA group than in HCs, which indicate that there are structural abnormalities in the MFG in individuals with CA. Meanwhile, we speculated that this abnormality may underlie working memory impairment in individuals with CA. There are two reasons for this speculation. The first reason is that many studies have reported that individuals with CA have deficits in working memory (27, 59–61). For instance, Hsieh et al. demonstrated that individuals with CA performed significantly worse in working memory tasks involving probed pitch recall (61). Jackson et al. also suggested that the short-term storage of pitch in working memory may be affected in CA (27). Sarkamo et al. also revealed that those with acquired amusia had more severe cognitive deficits than non-amusic patients, especially in working memory and executive functioning (62). The second reason for this speculation is that MFG is the core region underlying working memory (55). The MFG has been reported to be abnormally activated during working memory tasks in many patient populations, including patients with Parkinson's disease with mild cognitive impairment (63), major depressive disorder (64), and schizophrenia (54). Similarly, the MFG has also been reported to have strong activation in response to repeated pitch stimuli in individuals with CA, while the typical response to repeated stimuli is a reduction in brain activation, which suggests that individuals with CA may have deficits in attending to repeated pitch stimuli, or encoding repeated pitch stimuli into working memory (65). Our results showed that the mean fractal dimension value in the MFG was higher in the CA group than in HCs, indicating that there were structural abnormalities in the MFG in individuals with CA and may suggest that abnormalities of the MFG underlie working memory impairment in CA.

Moreover, we found significant decreases of sulcal depth in the right pars triangularis gyrus in the CA group compared with HCs, and a positive correlation between sulcal depth in the right pars triangularis gyrus and the mean MBEA score and subscores. These results revealed anatomical abnormalities in the IFG in patients with CA, and we assumed that this structural abnormality could represent the anatomical basis of mild speech perception impairment reported in those with CA. There are some reasons for this assumption. First, the right pars triangularis gyrus is located in the IFG and is known as the right homologue of Broca's area (66); it is an important structure for human language functions (66). Specifically, the IFG has been reported to be involved in phonological, syntactic, and semantic tasks such as tone and accent processing (67–69), sentence complexity (70), syntax processing (71), empathy (72), and emotional processing (73–75). For example, Chang et al. reported that the right IFG could be recruited in tone processing through its interaction with the right auditory cortex (67); Geiser et al. revealed that the right IFG played a specific task-related role in the processing of accent patterns (68). Additionally, Kotz et al. showed that activity in the right pars triangularis gyrus was activated when listening to prosodic speech compared to normal speech (73); Matsui et al. also demonstrated that the right pars triangularis gyrus was activated in the positive semantic content compared to negative prosody (75). Second, speech perception impairments in individuals with CA have been reported mainly on tone (19, 76), intonation (18, 20), and emotion (26, 77). For instance, Jiang et al. and Liu et al. all reported that individuals with CA showed impaired performance on tone identification and discrimination (18, 20). Cheung et al. found that patients with CA performed significantly less accurately than HCs in emotion prosody recognition (77); Thompson et al. reported that individuals with CA were significantly worse than HCs at decoding emotional prosody and these patients also reported difficulty understanding emotional prosody in their daily lives (26). Third, previous studies have reported that activation in the right IFG has been found during pitch processing in both non-linguistic (78) and speech contexts (79). Thus, the IFG abnormality in patients with CA may have implicit associations with acoustic pitch processing, resulting in the speech perception. Based on the above three points, we assumed that this IFG abnormality could represent the anatomical basis of mild speech perception impairment reported in those with CA.

Our correlation analysis also showed that fractal dimension in the MFG and the sulcal depth in the right pars triangularis gyrus were associated with the mean MBEA score. The MBEA has a good sensitivity and validity (7), is currently recognized as a diagnostic scale for CA, and is widely used in scientific research. However, it is worth noting that it takes a long time to complete, which could lead to patient fatigue and distracted attention even when there is catch trials and rest, in turn causing information biases (80). Additionally, the MBEA can be difficult for children with CA to complete. Peretz et al. reported that the MBEA was not useful for assessing children with 10 years of age, as the test length is excessive for children (7). More importantly, the MBEA does not encompass all perceptual skills, such as emotional appreciation, which may lead to some individuals with CA having deficits that the MBEA does not identify (7, 81). Thus, it could be useful to find a neurobiological marker of CA for an adjunct MBEA diagnosis. Our results revealed that cortical abnormalities in both the MFG and IFG were correlated with the MBEA score, which could demonstrate the feasibility and credibility of considering cortical morphological abnormalities in these two brain regions as a neurobiological marker of CA.

Finally, all the identified brain regions were located in the right hemisphere. In right-handed healthy humans, the right hemisphere is more specialized in musical and non-verbal processing, such as pitch discrimination, timbre detection, and musical structure processing, while the left hemisphere is more dedicated to linguistic and verbal processing, such as the processing of spoken words, digits, and syllables (82). CA mainly manifests as pitch perception and pitch memory impairments (5). Therefore, this could explain why cortical abnormalities were mainly found in the right hemisphere in the CA group in this study. In addition, speech perception impairment is common in CA, but we did not find abnormalities in the left hemisphere. This is probably because CA is a neurodevelopmental disorder and is involved in the abnormal neuronal migration and malformation (14, 83, 84). Therefore, we can speculate that there may be an abnormal migration of the language center from the left hemisphere to the right in those with amusia. This shift has been reported in patients with cerebral pathological entities, such as epilepsy (85), arteriovenous malformations (86), and brain tumors (87); however, in neurodevelopmental disorders, this hypothesis should be confirmed in future studies. Furthermore, many structural and fMRI studies have focused on abnormalities in the right hemisphere (10, 87). Our study supports previous findings, suggesting that CA is a disorder that is associated with abnormalities in the right hemisphere of the brain.

### Limitations

Despite the novel discoveries of the present study, it has some limitations that should be noted. First, the final sample from which structural MRI data were obtained was relatively small due to the rarity of the disease. Given that our study had a small sample size, we focused on detecting structural abnormities, but did not investigate the link between the right MFG and right pars triangularis gyrus, and did not evaluate the relationship between scores of a detailed language and memory scale and abnormal brain anatomy. Future work should use larger sample sizes, refine behavioral materials, and explore the relationship between behavior and neurobiology in pitch disorders.

## Conclusions

Our findings indicate that CA is associated with cortical morphological changes. Our findings may suggest that the neural basis of speech perception and memory impairments in those with CA is associated with abnormality in the right pars triangularis gyrus and MFG, and that the cortical abnormality in these two brain regions may represent a neural marker of CA.

## Data Availability Statement

The original contributions presented in the study are included in the article/supplementary material, further inquiries can be directed to the corresponding author/s.

## Ethics Statement

The studies involving human participants were reviewed and approved by the Ethics Committee of the Second Xiangya Hospital, Central South University. Written informed consent to participate in this study was provided by the participants' legal guardian/next of kin.

## Author Contributions

DW, ZJ, and JL designed the project. ZJ, JS, and XL collected the data. Data analysis and manuscript writing were conducted by XL. The manuscript revision was conducted by XL and JL. All authors approved the final version of the manuscript.

## Funding

This work was supported by the National Natural Science Foundation of China (Grant No. 81771172, to DW), the Clinical Research Center for Medical Imaging in Hunan Province (Grant No. 2020SK4001).

## Conflict of Interest

The authors declare that the research was conducted in the absence of any commercial or financial relationships that could be construed as a potential conflict of interest.

## Publisher's Note

All claims expressed in this article are solely those of the authors and do not necessarily represent those of their affiliated organizations, or those of the publisher, the editors and the reviewers. Any product that may be evaluated in this article, or claim that may be made by its manufacturer, is not guaranteed or endorsed by the publisher.
